# Complement Mediated Signaling on Pulmonary CD103^+^ Dendritic Cells Is Critical for Their Migratory Function in Response to Influenza Infection

**DOI:** 10.1371/journal.ppat.1003115

**Published:** 2013-01-10

**Authors:** Matheswaran Kandasamy, Poon C. Ying, Adrian W. S. Ho, Hermi R. Sumatoh, Andreas Schlitzer, Timothy R. Hughes, David M. Kemeny, B. Paul Morgan, Florent Ginhoux, Baalasubramanian Sivasankar

**Affiliations:** 1 Infection and Immunity Programme, Singapore Institute for Clinical Sciences, Agency for Science, Technology and Research (A*STAR), Singapore; 2 Singapore Immunology Network (SIgN), Agency for Science, Technology and Research (A*STAR), Singapore; 3 Institute of Infection and Immunity, School of Medicine, Cardiff University, Cardiff, Wales, United Kingdom; 4 Immunology Programme and Department of Microbiology, National University of Singapore, Singapore; Johns Hopkins University - Bloomberg School of Public Health, United States of America

## Abstract

Trafficking of lung dendritic cells (DCs) to the draining lymph node (dLN) is a crucial step for the initiation of T cell responses upon pathogen challenge. However, little is known about the factors that regulate lung DC migration to the dLN. In this study, using a model of influenza infection, we demonstrate that complement component C3 is critically required for efficient emigration of DCs from the lung to the dLN. C3 deficiency affect lung DC-mediated viral antigen transport to the dLN, resulting in severely compromised priming of virus-specific T cell responses. Consequently, C3-deficient mice lack effector T cell response in the lungs that affected viral clearance and survival. We further show that direct signaling by C3a and C5a through C3aR and C5aR respectively expressed on lung DCs is required for their efficient trafficking. However, among lung DCs, only CD103^+^ DCs make a significant contribution to lung C5a levels and exclusively produce high levels of C3 and C5 during influenza infection. Collectively, our findings show that complement has a profound impact on immune regulation by controlling tissue DC trafficking and highlights a potential utility for complement as an adjuvant in novel vaccine strategies.

## Author Summary

Influenza is a global health problem frequented by epidemics and pandemics. Current vaccines against influenza offer limited protection hence the need for reformulation and repeated vaccination. There is a pressing need to develop newer vaccines that are able to generate T cell response. In order to develop such vaccines, there is a need to understand how T cell responses are generated during influenza infection. Influenza specific T cell responses are generated by the dendritic cells (DCs) in the lung. Upon influenza infection, DCs in the lung carry viral peptides to the draining lymph node (dLN) to initiate an immune response. Thus, migration of DCs from the lung to the dLN is an important step in the initiation of influenza specific T cell response. We now show that activation products of the complement system interact with their receptors on the DCs, which signals for the DCs to migrate from the lung to the dLN. Thus, our results reveal a previously unknown function for complement in mediating lung DC migration during influenza infection and highlight its potential as an adjuvant in novel vaccine strategies.

## Introduction

Influenza is a global health problem and current vaccination strategies are still inadequate at providing protection against seasonal and epidemic outbreaks [1]. Vaccination strategies aiming to induce a protective CD8^+^ T cell response hold tremendous potential, since CD8^+^ T cells are able to recognize core epitopes conserved across a wide range of influenza strains [2], [3]. Hence, there is a pressing need to improve our understanding on the mechanisms that contribute to the orchestration of CD8^+^ T cell responses during influenza infection.

Influenza-specific T cell responses are initiated and maintained by lung dendritic cells (DCs) which are strategically localized within the respiratory tract to mediate this process effectively [4], [5]. DCs comprise a heterogeneous population of antigen sensing and presenting cells that control the initiation of T cell responses thus bridging innate and adaptive immune responses [6]. Different subsets of DCs with unique homeostasis and immune functions had been described in both lymphoid and non-lymphoid tissues [7]. In this regard, lung resident DCs can be divided into CD103^+^CD11b^−^ (CD103^+^ DCs) and CD103^−^CD11b^+^ (CD11b^+^ DCs) based on the expression of the integrins α_E_β_7_ (CD103) and ITGAM (CD11b) respectively [8], [9]. During influenza infection, both types of lung resident DCs migrate to the dLN to prime T cells [10] henceforth referred to as migratory DC subsets (mDCs).

The complement system is an essential component of the innate immune network and has evolved as an important bridge between innate and adaptive immune systems, similarly to DCs, but at the molecular level [11], [12], [13], [14]. In particular, complement component C3 has been shown to impact antiviral T cell immunity and allograft rejection in a mechanism independent of intrinsic C3 expression in T cells [15], [16], [17], [18]. These observations suggested the involvement of an additional cell type expressing C3 that control T cell activation. Recent studies using bone marrow derived DCs have revealed that C3 is essential for DCs to efficiently stimulate T cells [19], [20], suggesting that DCs mediate these aforementioned complement effects [15], [16], [17] on T cells by acting both as complement producing and sensing cells. However, the *in vivo* functional relevance of C3 in peripheral tissue DCs, which play a central role in the induction of immunity by virtue of their location [21], remains largely unexplored, necessitating further studies.

Influenza infection is known to activate complement both locally in the lung and systemically [22], [23], but the biological significance of complement activation during influenza infection remains poorly understood. The functional importance of C3 in T cell immunity during influenza infection was initially demonstrated by Kopf et al., who showed that C3^−/−^ mice exhibited reduced T cell response to influenza infection and attributed this to a possible priming defect mediated by DCs [16]. Thus, we hypothesized that C3 may be critically involved in the control of the T cell priming function of lung mDCs during influenza infection.

In this study, we used C3-deficient (C3^−/−^) mice to show that C3 was critical for survival during influenza infection, and C3 deficiency was associated with attenuated T cell priming in the dLN and reduced development of effector T cell responses in the lung as previously shown [16]. However, we found that this defective priming was not due to altered priming ability of C3-deficient DCs but rather due to a defect of mDC migration from the lung to the dLN in C3^−/−^ mice. We further demonstrated that the direct interaction of complement activation products C3a and C5a with their receptors expressed on the surface of mDCs was critical for their migration. Finally, we identified CD103^+^ DCs as the sole mDC subset capable of secreting C3 and C5 and one of the major source of lung C5a during influenza infection. Altogether, our results establish a previously unidentified role for complement activation products C3a and C5a in mediating migratory function of mDCs and highlight the crucial role of CD103^+^ DC subset as an unique complement sensing and producing DC population controlling its own migration and the migration of CD11b^+^ DCs.

## Results

### Complement component C3 is critical for survival during influenza infection

**Figure 1:**
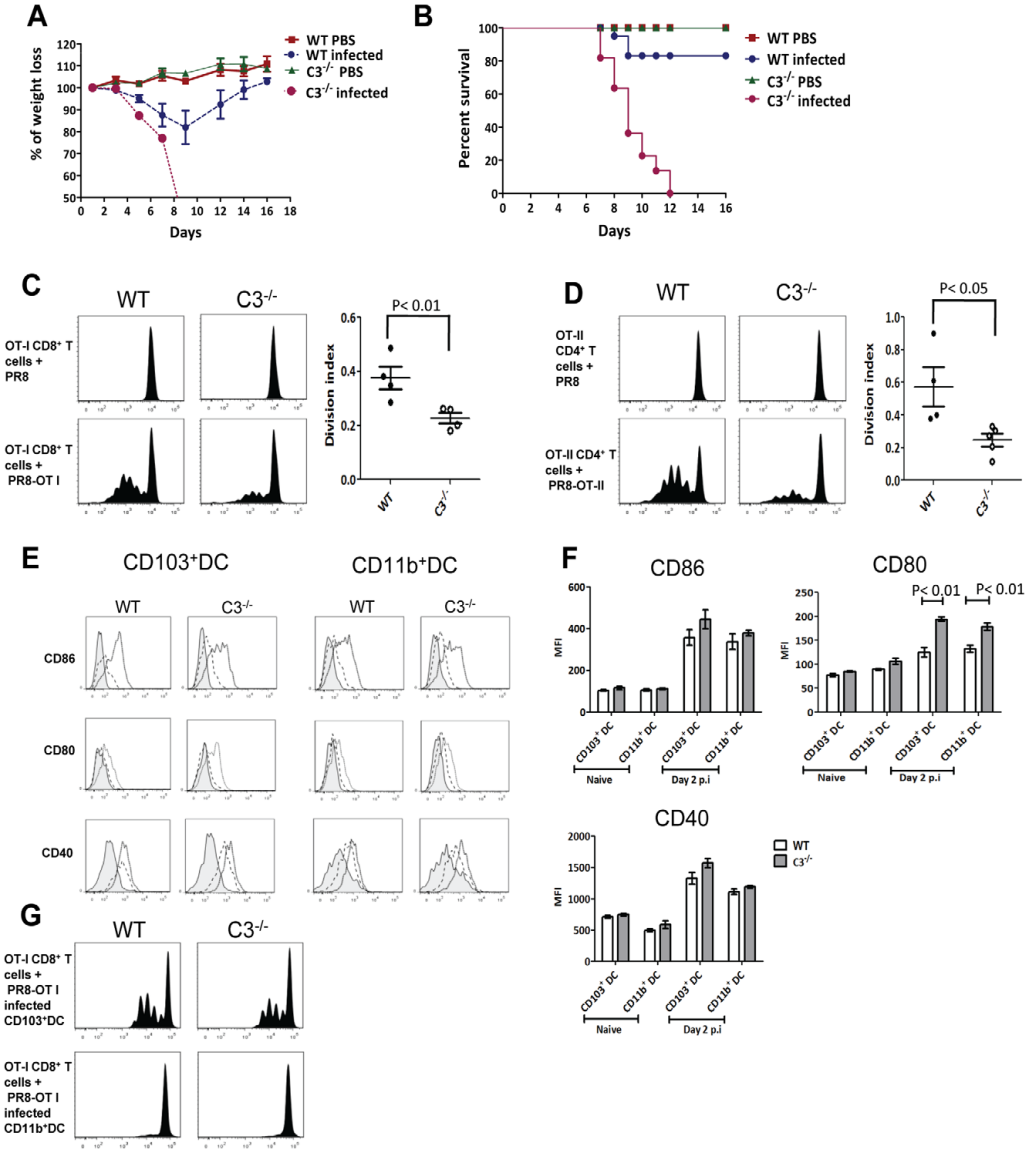
C3^−/−^ mice show greater weight loss and mortality during influenza infection. (**A**) Percentage of body weight loss after influenza infection. A weight loss of <20% and recovery represents a sub-lethal infection. (**B**) Survival curve comparing WT and C3^−/−^ mice during influenza infection (n = 6–7 in each group). (**C**) Plots represent *ex vivo* analysis of CFSE-labeled OT-I CD8^+^ T cell proliferation in the dLN 3 days after infection with PR8 and PR8-OT-I (bottom). The graph on the right shows the respective division index for the proliferating OT-I CD8^+^ T cells for each group of mice. (**D**) Plots represent *ex vivo* analysis of CFSE-labeled OT-II CD4^+^ T cell proliferation in the dLN 3 days after infection with PR8 and PR8-OT-II (bottom). The graph on the right shows the respective division index for the proliferating OT-II CD4^+^ T cells for each group of mice. (**E**) Histogram showing expression of costimulatory molecules CD86, CD80 and CD40 on CD103^+^ DCs and CD11b^+^ DCs in the dLN on day 2 post infection. Filled histogram: Isotype control, dashed histogram: Naïve, open histogram: infected. (**F**) Bar graphs show the mean fluorescent intensity (MFI) of CD86, CD80 and CD40 expression on CD103^+^DC and CD11b^+^ DC subsets in the dLN of naïve and influenza infected (Day 2) WT or C3^−/−^ mice. (**G**) Plots represent CFSE-labeled OT-I CD8^+^ T cell proliferation 3 days after co-culture with sorted CD103^+^ DCs and CD11b^+^ DCs obtained from pooled dLN of PR8-OT-I influenza infected mice at a ratio of 1∶10 (DC:T cells). Results shown are representative of four experiments with similar results. The values are expressed as mean ± SEM.

C3 has been shown to be important for eliciting T cell responses and viral clearance during influenza infection in mice [16], although the impact of C3 deficiency on survival after influenza infection was not well reported. In order to evaluate this, we infected WT and C3^−/−^ mice with a sub-lethal dose (optimized in WT mice, 15 PFU for female and 25 PFU for male) of influenza virus and monitored their weight loss and survival. WT mice showed ∼20% weight loss at the peak of infection and recovered with 20% mortality as previously reported [24]. In stark contrast, C3^−/−^ mice showed greater weight loss on days 5 and 7, and resulted in 100% mortality by day 12 (Fig. 1A and B). Subsequent evaluation of effector T cell responses and viral load in the lungs recapitulated previous observations [16], showing decreased effector T cell responses and viral clearance in the C3^−/−^ mice (Fig. S1).

Absence of C3 in T cells does not directly alter their function [16], hence we reasoned that these defects in T cell responses were mediated by the lack of C3 in DCs, as suggested in previous reports [19], [20]. In order to investigate whether priming of antigen-specific T cells in the dLN in response to influenza infection was altered in C3^−/−^ mice, we adoptively transferred CFSE labeled OT-I CD8^+^ transgenic T cells into mice prior to infection with either wild type PR8 influenza virus or recombinant PR8 influenza virus containing the OVA epitope SIINFEKL (PR8-OT-I). We then harvested lung dLNs at indicated time points post-infection to assess proliferation by CFSE dilution on SIINFEKL-K^b^ tetramer positive CD8^+^ T cells. As expected, proliferation was observed only when mice were inoculated with PR8-OT-I, and no proliferation was observed with PR8 influenza virus alone, reflecting the specificity of the response (Fig. 1C). Strikingly, the level of proliferation was significantly reduced in the C3^−/−^ mice in comparison with WT mice (Fig. 1C). CD4^+^ T cell priming in the dLN was also evaluated using OT-II (CD4) transgenic T cells and PR8 influenza virus containing the OVA epitope ISQAVHAAHAEINEAGR (PR8-OT-II). Similarly, we found that the priming of CD4^+^ T cells was significantly reduced in the C3^−/−^ mice when compared to WT controls (Fig. 1D).

Since C3 has been reported to influence the expression of co-stimulatory molecules on DCs [19], we tested whether diminished levels of costimulation could explain the decreased T cell responses by measuring the expression of costimulatory molecules on mDCs in the dLN of both WT and C3^−/−^ mice on day 2 post-infection. Resident and mDC subsets in the dLN were characterized as shown in Fig. S2. We found comparable levels of CD86 and CD40 expression, while CD80 expression was even increased in the C3^−/−^ mDCs (Fig. 1E and F), excluding an intrinsic maturation defect in C3^−/−^ DCs. Finally, to understand whether the T cell priming defect observed *in vivo* was due to a decreased priming ability by C3^−/−^ mDCs (Fig. 1C and D), we sorted CD103^+^ DCs and CD11b^+^ DCs on day 2 post infection with PR8-OT-I and evaluated their priming ability *ex vivo*. A strong proliferation of OT-I CD8^+^ T cells was observed only when CD8^+^ T cells were co-cultured with CD103^+^ DCs, but not with CD11b^+^ DCs, an observation supporting the predominant role of CD103^+^ DCs in early CD8^+^ T cell priming [10]. However, *ex vivo* priming ability was comparable between WT and C3-deficient CD103^+^ DCs (Fig. 1G), demonstrating that there was no intrinsic priming defect in C3-deficient CD103^+^ DCs.

### C3^−/−^ mice show defective trafficking of mDC subsets from the lung to the dLN during influenza infection

**Figure 2:**
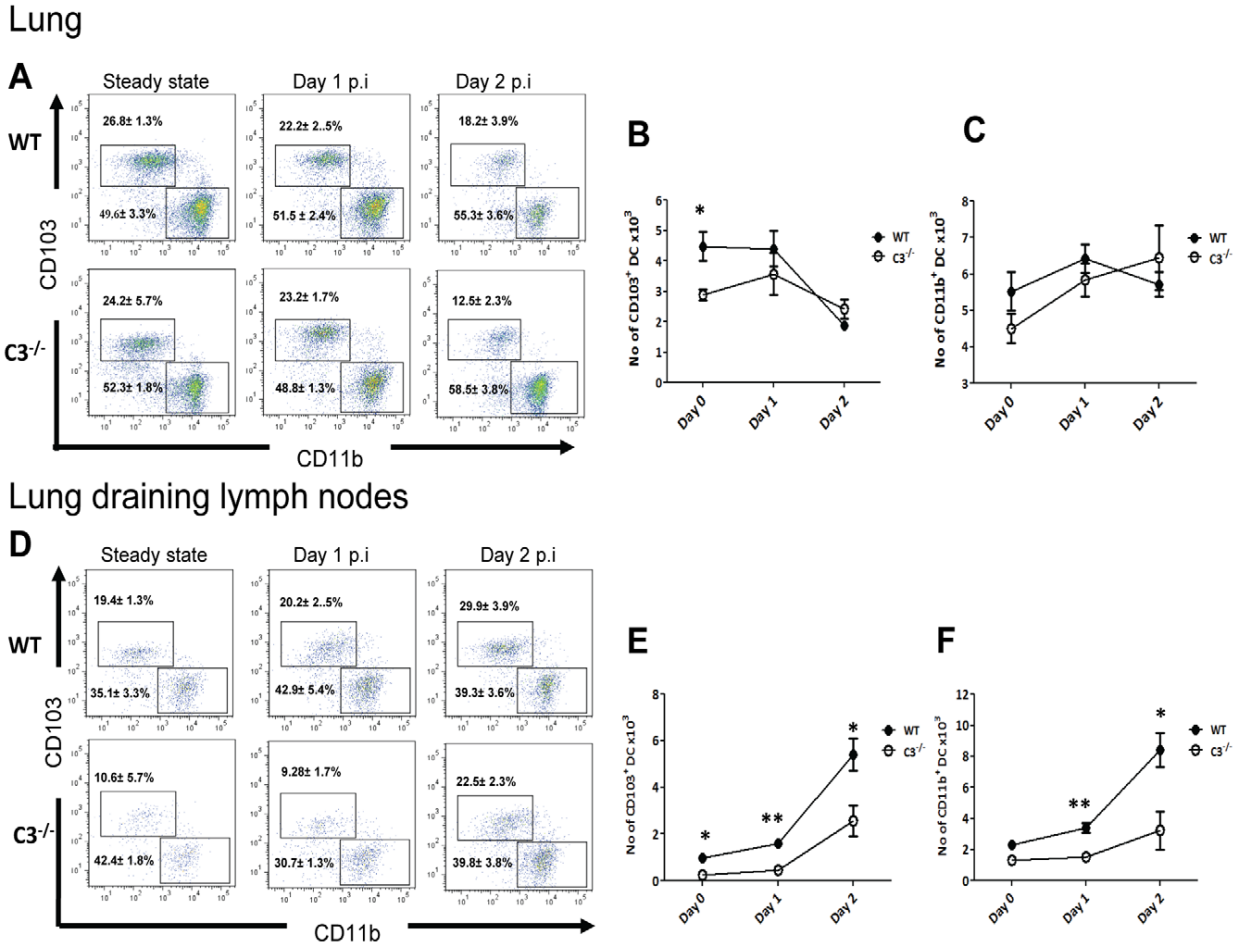
Migratory DC subsets are reduced in the dLN in C3^−/−^ mice after influenza infection. (**A**) Kinetics of CD103^+^ DCs and CD11b^+^ DCs in the lungs during influenza infection in WT and C3^−/−^ mice. Populations were gated as show in Fig. S2. Numbers within the dot plot represent average percentage of cells for the group. (**B** and **C**) Absolute numbers of CD103^+^ DC (**B**) and CD11b^+^ DC (**C**) subsets in lungs during steady state and during influenza infection. (**D**) Kinetics of CD103^+^ DCs and CD11b^+^ DCs in the dLN during influenza infection in WT and C3^−/−^ mice. Populations were gated as shown in Fig. S1. Numbers within the dot plot represent average percentage of cells for the group. (**E** and **F**) Absolute numbers of CD103^+^ DC (**E**) and CD11b^+^ DC (**F**) subsets in dLN during steady state and during influenza infection. The values are expressed as mean ± SEM. The data are representative of three different experiments with similar results. *, P<0.05, and **P<0.01.

Next, we investigated the composition of mDC subsets in the lung and lung dLN at steady state and during the course of influenza infection to test whether the priming defect could be explained by a defect of the mDC network in the lung and dLN of C3^−/−^ mice. The gating strategy employed for characterizing lung mDCs is shown in Fig. S3 [25], [26]. Under steady state conditions, the relative numbers of CD103^+^ DCs and CD11b^+^ DCs were comparable between C3^−/−^ and WT mice, whereas enumeration of absolute numbers showed a slight decrease for CD103^+^ DCs only in the C3^−/−^ mice (Fig. 2A–C). Upon influenza infection, both the relative and absolute numbers of lung CD103^+^ DCs and CD11b^+^ DCs became comparable between C3^−/−^ and WT mice during influenza infection (Fig. 2A–C). These results show that the T cell priming deficit observed in C3^−/−^ mice is not due to a defect in the differentiation of lung mDCs.

Next, we assessed whether the migration ability of lung mDCs was compromised. Under steady state, lung mDCs constitutively migrate at a slow rate to the dLN, a process strongly increased under inflammatory conditions [8]. As expected, in WT mice during the course of infection, the absolute numbers of both CD103^+^ DCs and CD11b^+^ DCs increased in the dLN by day 1 post infection and further by day 2 post infection (Fig. 2D–F). However, absolute numbers of both CD103^+^ DCs and CD11b^+^ DCs were significantly decreased in C3^−/−^ mice, suggesting a decreased migration capacity of CD103^+^ DCs and CD11b^+^ DCs at both steady state and at all time points tested after influenza infection (Fig. 2D–F).

**Figure 3:**
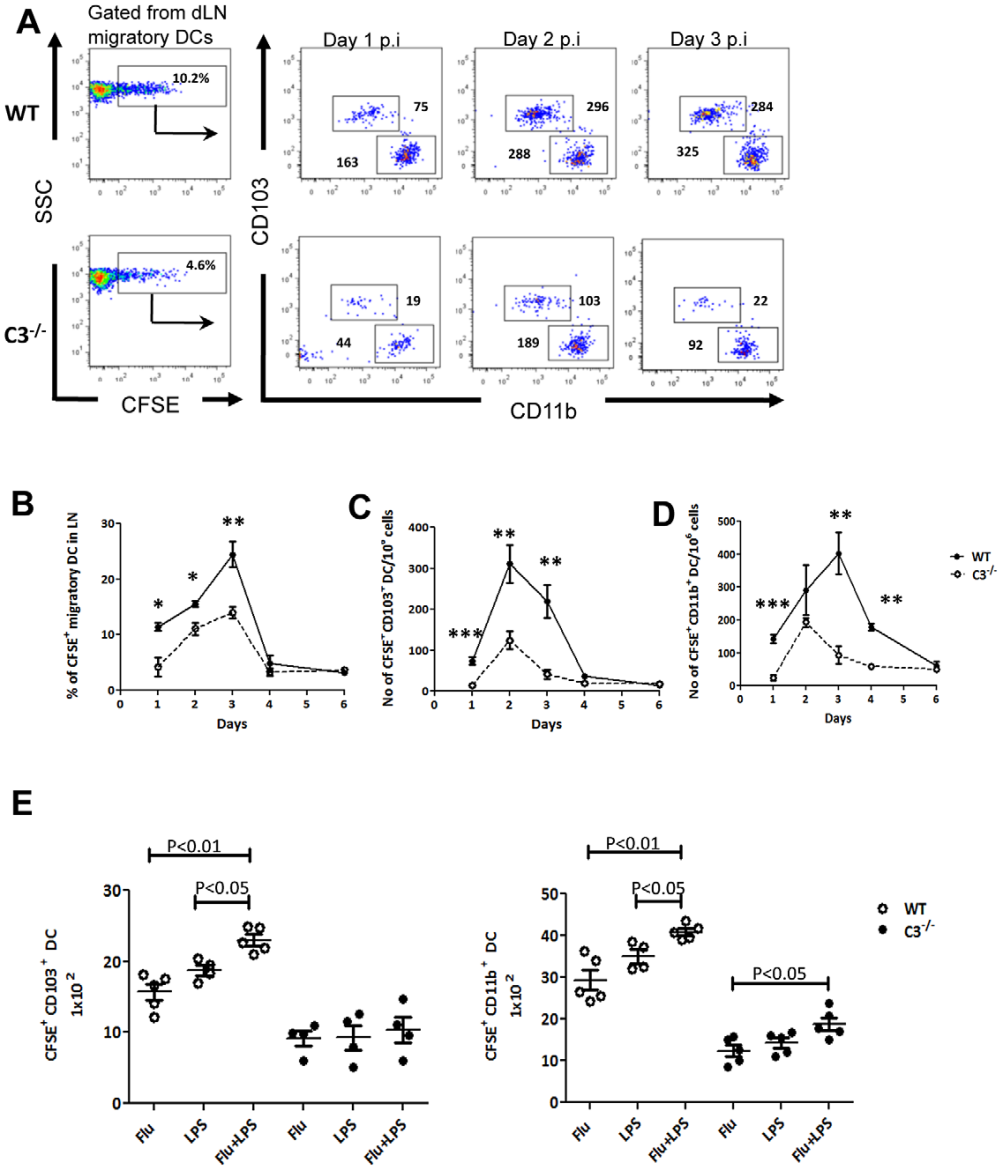
Tracking of mDC migration from the lung to the dLN demonstrates defective mDC migration in C3^−/−^ mice during influenza infection and intratracheal administration of LPS does not recover the defective mDC migration during influenza infection. WT and C3^−/−^ mice were infected with influenza virus; 16 hours before sacrificing, cells in the lungs were labeled with 8 mM CFSE by intranasal instillation. dLN was harvested and analysed for CFSE^+^ mDCs using gating strategy shown in Fig. S1. (**A**) Dot plots show CFSE^+^ mDCs (first panel) in the dLN and subsequent panels show CD103^+^ DCs and CD11b^+^ DCs after gating on CFSE^+^ mDCs at indicated times after influenza infection. Numbers within the dot plot represent cell number. (**B**) Kinetics of frequency of CFSE^+^ mDCs in the dLN of influenza infected mice. Absolute number of CFSE^+^ CD103^+^ DCs (**C**) and CFSE^+^ CD11b^+^ DCs (**D**) in the dLN at indicated time points after influenza infection. LPS (2 µg in PBS) was administered intratracheally after 6 hours to either uninfected or flu infected C3^−/−^ mice and the migration of mDCs from the lung to the dLN were followed as described earlier using CFSE on day 2 post infection. (E) Number of CFSE^+^ CD103^+^ (left) and CD11b^+^ (right) DCs in LPS administered C3^−/−^ mice in comparison with WT mice without LPS administration. The values are expressed as mean ± SEM. The data are representative of three different experiments with similar results. *, P<0.05, ** P<0.01 and *** P<0.001.

To confirm these data, the migration of mDCs from the lung to the dLN was tracked using CFSE delivered intranasally into the lungs at different time points after influenza infection [27]. As expected, the relative numbers of CFSE^+^ mDCs in the dLN increased after influenza infection, reaching a maximum by day 3 in the WT mice. However, CFSE^+^ mDC accumulation (independent of subsets) was significantly reduced in the C3^−/−^ dLN (Fig. 3A left panel and B). Upon enumeration of CD103^+^ DCs and CD11b^+^ DCs within the CFSE^+^ mDC population, we found that migration of both CD103^+^ DCs and CD11b^+^ DCs was severely compromised in the C3^−/−^ mice (Fig. 3A, C and D). In order to understand whether a strong inflammatory signal is able to overcome the defective signaling on mDCs due to the lack of C3, we administered LPS intratracheally shortly after influenza infection and then evaluated mDC migration in C3^−/−^ mice. Our observations indicated that LPS administration was not able to overcome the defective trafficking of both subset of lung mDCs in the C3^−/−^ mice, although a mild effect was observed for CD11b^+^ DCs (Fig. 3E). These observations indicate that the critical importance of complement mediated signaling in lung mDC trafficking is independent of inflammatory signals. Altogether, our observations demonstrate that migration of C3^−/−^ mDCs to the dLN is significantly reduced compared to WT during inflammation.

### Viral uptake and maturation is not affected in mDCs from C3^−/−^ mice

**Figure 4:**
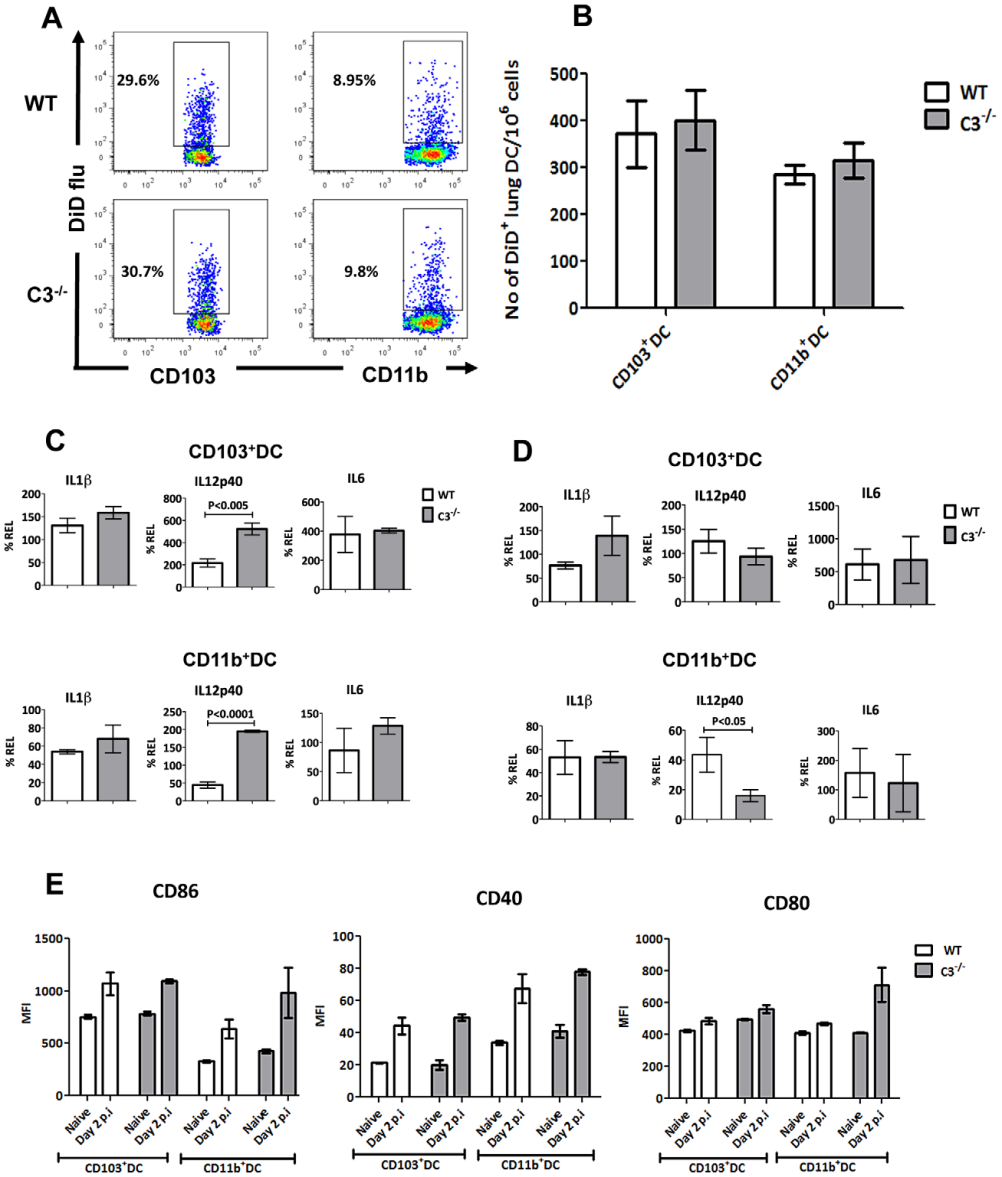
C3^−/−^ mDCs display similar capacity for virus uptake and maturation. (**A**) Dot plots show the percentage of DiD flu^+^ uptake by CD103^+^ DC (left panel) and CD11b^+^ DC (right panel) in WT and C3^−/−^ mice 16 hrs after DiD flu administration on day 1 post infection with PR8. (**B**) Bar graph shows the average numbers of DiD flu^+^ CD103^+^ DCs and DiD flu^+^ CD11b^+^ DCs in the lungs of WT and C3^−/−^ mice. (**C**)Single cell preparations from the lungs were enriched for DCs by a density gradient method. Enriched DCs were infected with influenza virus under *ex vivo* culture conditions and six hours later the CD103^+^ and CD11b^+^ DCs were flow sorted. Q-RT-PCR for the indicated cytokines were performed on the RNA obtained from the CD103^+^ and CD11b^+^ DC populations. Bar graph shows % relative expression in comparison to un-infected control. (**D**) WT and C3^−/−^ mice were infected with flu and 24 hours later the CD103^+^ and CD11b^+^ DCs from the lungs were flow sorted. Q-RT-PCR for the indicated cytokines were performed on the RNA obtained from the CD103^+^ and CD11b^+^ DC populations. Bar graph shows % relative expression in comparison to un-infected control. (**E**) Bar graphs shows the mean fluorescent intensity of CD86, CD40 and CD80 expression on CD103^+^DC and CD11b^+^ DC subsets in the lungs of naïve and PR8 infected WT or C3^−/−^ mice. The data are representative of three different experiments with similar results. The values are expressed as mean ± SEM.

Complement component C3 mediated opsonization has been shown to affect viral uptake by DCs [28]. To test whether reduced viral uptake by C3^−/−^ DCs could explain the defective mDCs migration in C3^−/−^ mice, we examined their antigen uptake capacity using DiD labeled influenza virus as previously described [25]. We first infected mice with unlabelled PR8 influenza virus to establish an infection and its associated inflammation, and subsequently inoculated the same mice with DiD labeled PR8 influenza virus 16 hours before harvesting the lungs. DiD^+^ mDC subsets were comparable between C3^−/−^ and WT mice suggesting that the lack of C3 did not affect antigen uptake (Fig. 4A and B).

Migratory DCs upon exposure to antigen under inflammatory conditions undergo maturation before migrating to the dLN and C3 is known to influence the expression of costimulatory molecules [29]. In order to understand whether the mDCs in the C3^−/−^ mice produced sufficient inflammatory mediators comparable to the WT mice, we examined the expression levels of inflammatory cytokines in the mDCs after *ex vivo* and *in vivo* influenza infection. Expression levels of IL-1β and IL-6 were comparable in the mDCs under both *ex vivo* and *in vivo* conditions. However IL-12p40 expression were higher in the C3^−/−^ mice when mDCs were infected under *ex vivo* conditions whereas comparable under in vivo conditions. (Fig. 4C and D). Subsequently, we evaluated the maturation of mDC subsets by analyzing the expression of co-stimulatory molecules after influenza infection (Fig. 4E). Expression levels of CD86, CD80 and CD40 on mDCs were comparable between C3^−/−^ and WT mice during the course of infection.

**Figure 5:**
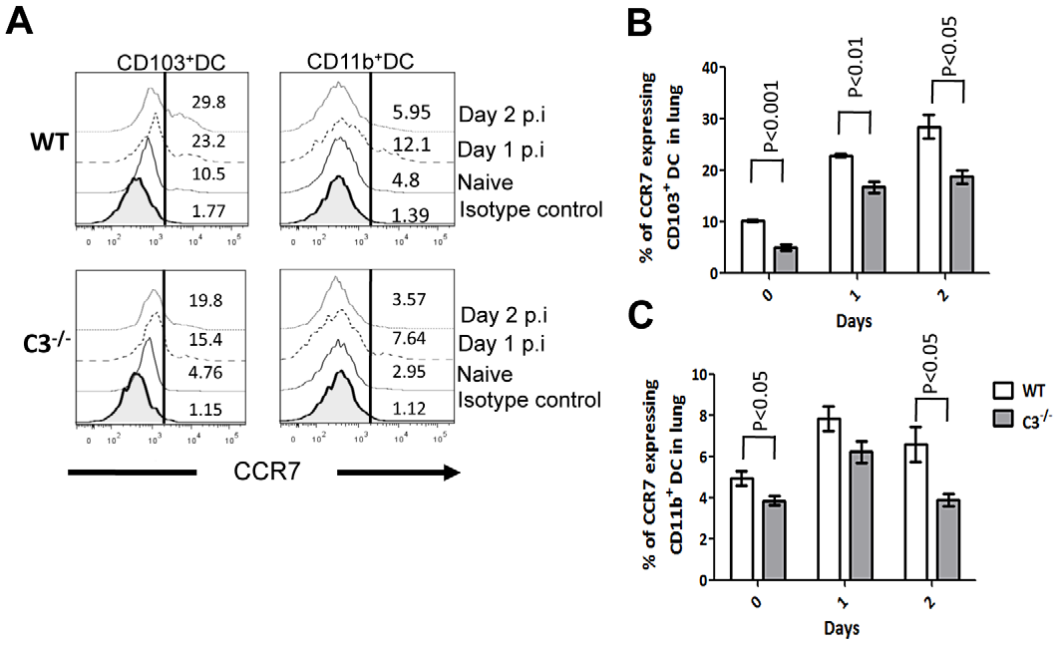
C3^−/−^ mDCs show similar fold CCR7 up regulation upon influenza infection. (**A**) Representative FACS plots for CCR7 expression in lung mDC subsets of naïve and PR8 infected WT or C3^−/−^ mice. Percentage CCR7 expression on CD103^+^ DCs (**B**) and CD11b^+^ DCs (**C**) during the course of influenza infection. The values are expressed as mean ± SEM.

CCR7 expression on mDCs has been shown to be important for their migration to dLN [29], [30], [31], hence we evaluated the expression of CCR7^+^ at the surface of lung mDCs. Under steady state, the frequency of CCR7 expressing mDCs was lower in C3^−/−^ mice (Fig. 5A–C). After influenza infection, the relative numbers of CCR7 expressing mDCs increased in both groups to a similar extent. However due to the reduced initial frequency of CCR7^+^ mDCs in C3^−/−^ mice at steady state, the proportion of CCR7^+^ mDCs upon flu infection remained lower than WT (Fig. 5A–C). These observations suggest that lack of C3 did not affect the up- regulation of CCR7 expression on mDCs under inflammatory conditions.

Altogether, these results show that C3-deficient mDCs are not defective in viral uptake and are fully competent in the expression of inflammatory mediators and co-stimulatory molecules for T-cell priming, but are less migratory.

### Complement components C3 and C5 are produced by CD103^+^ DCs upon influenza infection

**Figure 6:**
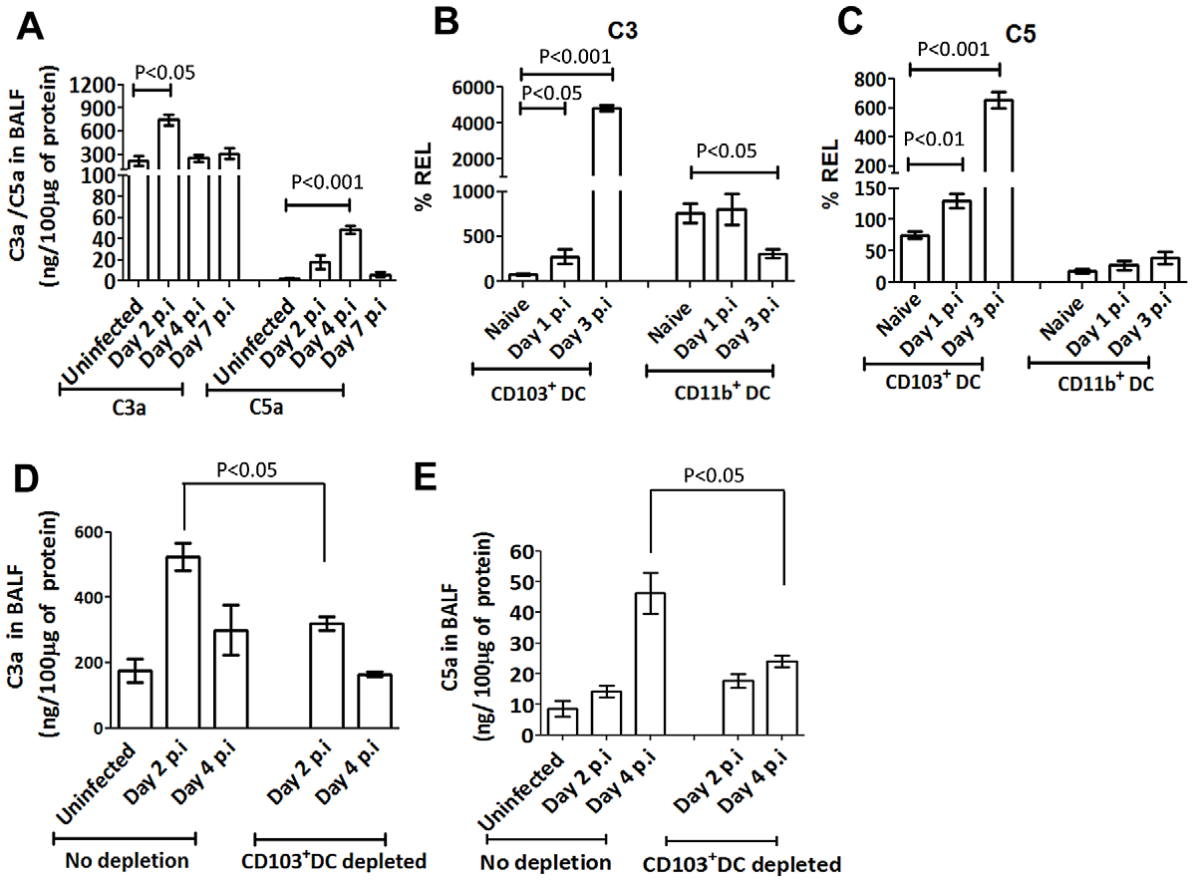
Lung C3a and C5a levels are up regulated during influenza infection by exclusive contribution from CD103^+^ DCs. (**A**) Bar graph shows the amounts of C3a and C5a per 100 µg protein in BAL fluid from naïve and PR8 infected WT mice on day 2, 4 and 7 post infection. (**B** and **C**) Bar graphs showing the relative mRNA expression levels of C3 and C5 in FACS sorted lung CD103^+^ DC and CD11b^+^ DC subsets in naïve or PR8 infected WT mice on day 1 and 3 post infection. Highest expression level in the naïve mice is taken as 100%. (**D** and **E**) Bar graphs show the amount of C3a and C5a per 100 µg protein in BAL fluid from naïve and PR8 infected langerin DTR mice with or without the depletion of CD103^+^ DCs. The data are representative of three different experiments with similar results. The values are expressed as mean ± SEM.

The absence of C3 causes a complete block in complement activation and hence C3^−/−^ mice lack the ability to generate both C3a and C5a [22]. To ascertain whether these activation products are directly involved in mediating mDC migration, we first quantified the levels of C3a and C5a in the bronchioalveolar lavage fluid before and after influenza infection in WT mice by ELISA. At steady state, C3a levels were high, further increasing by approximately 4-fold on day 2 after infection and returning to background level by day 4 (Fig. 6A). In the case of C5a, steady state levels were very low, increasing by approximately 7-fold and 17-fold on days 2 and 4 respectively post infection, before returning to background levels by day 7 post infection (Fig. 6A).

Next, we investigated the cellular source of C3a and C5a and assessed the ability of lung mDCs to synthesize and secrete C3 and C5 during infection. Lung DCs before and after influenza infection were sorted and C3 and C5 mRNA expression levels were determined by qRT-PCR. Both C3 and C5 mRNA levels were significantly upregulated after influenza infection in CD103^+^ DCs; C3 levels were upregulated by approximately 3-fold on day 1 post infection and 50-fold on day 3 after infection (Fig. 6B), while C5 levels were upregulated 2-fold on day 1 post infection and 5-fold on day 3 (Fig. 6C). No modulation of C3 and C5 mRNA expression was observed in CD11b^+^ DCs after influenza infection (Fig. 6B and C).

Since only CD103^+^ DCs showed increased C3 and C5 mRNA expression upon influenza infection, we evaluated the contribution of CD103^+^ DCs to the observed increase in C3a and C5a levels in the lung during influenza infection. For this purpose, we used langerin-DTR mice to specifically deplete CD103^+^ DCs [10]. Langerin-DTR mice specifically expressed DTR on CD103^+^ DCs in the lung and DT administration efficiently depleted lung CD103^+^ DCs (Fig. S4), and subsequent influenza infection in DT-treated langerin-DTR mice did not cause increased C3a and C5a levels in the lungs on days 2 and 4 when compared with CD103^+^ DCs sufficient mice (Fig. 6D and E).

**Figure 7:**
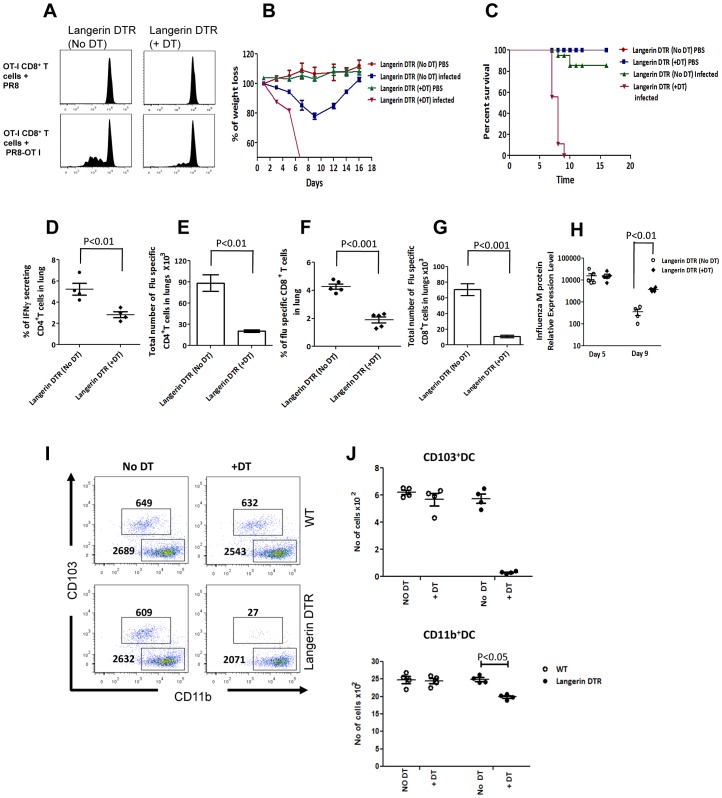
Langerin-DTR mice show effector T cells response and survival characteristics similar to those of C3^−/−^ mice upon influenza infection and has defective CD11b^+^ DC migration. (**A**) Plots represent *ex vivo* analysis of CFSE-labeled OT-I CD8^+^ T cells proliferation in the dLN 3 days after infection with PR8 and PR8-OT-I (bottom) in WT and CD103^+^ DCs depleted langerin-DTR mice. (**B**) Percentage of body weight loss after influenza infection. (**C**) Survival curve comparing WT, C3^−/−^ and Langerin –DTR mice during influenza infection (n = 6 per group). (**D**) Graph shows the relative mRNA expression level of influenza M protein in lung tissues of WT and CD103^+^ DCs depleted langerin-DTR mice infected with 4 PFU of PR8. (**E**) Graph shows the frequency of IFNγ secreting CD4^+^ T cells in lungs by *ex vivo* overnight stimulation with MHC-II flu peptide on day 7 post infection. (**F**) Bar graph shows the absolute numbers of IFNγ secreting CD4^+^ T cells in lungs on day 7 post infection (**G**) Graph shows the frequency of Flu specific CTL response in lung as measured by Flu _peptide_ (ASNENMETM (NP _366–374_)/H-2D^b^ tetramer staining on day 7 post infection. (**H**) Bar graph shows the absolute numbers of flu specific CD8^+^ T cells in lungs by tetramer staining on day 7 post infection. DT treated WT and Langerin-DTR mice were flu infected and the migration of CD103^+^ and CD11b^+^ DCs were evaluated as described before on day 3 post infection. (**I**) Dot plots show CD103^+^ and CD11b^+^ DCs mDCs in the dLN of DT treated and untreated WT and Langerin-DTR mice. Numbers within the dot plot represent cell number. (**J**) Total number of CD103^+^(top) and CD11b^+^(bottom) DCs in the dLN of flu infected mice. Results shown are representative of at least three different experiments with similar results. The values are expressed as mean ± SEM.

Importantly, when depletion of lung CD103^+^ DCs in langerin-DTR mice was followed by infection with PR8-OT-I, priming of OT-I CD8^+^ T cells in the dLN was severely reduced when compared with PR8-OT-I infected WT (Fig. 7A), similar to our observations in the C3^−/−^ mice (Fig. 1C). CD103^+^ DCs depleted langerin-DTR mice infected with influenza displayed greater weight loss (Fig. 7B), higher mortality (Fig. 7C), reduced lung effector T cell response (Fig. 7D–G) and increased viral load (Fig. 7H) as compared to WT, paralleling our observations in C3^−/−^mice (Fig. 1A and B). Since DT administered langerin-DTR mice showed rapid mortality, we evaluated whether DT administration induced any toxicity during influenza infection in WT mice. Our observation did not indicate any toxicity for DT in WT mice during influenza infection (Fig. S5).

Among the mDC subsets, only the CD103^+^ DCs were found to produce the complement components C3 and C5 upon influenza infection (Fig. 6B and C) and depletion of CD103^+^ DCs significantly reduced the availability of C3a and C5a in the lungs (Fig. 6D and E). We hypothesized that CD11b^+^ DCs rely on complement produced by CD103^+^ DCs for their migration to the dLN. Therefore, we followed the migration of CD11b^+^ DCs in the langerin-DTR mice after depleting the CD103^+^ DCs. Our results suggested that depletion of CD103^+^ DCs in langerin-DTR mice significantly affected the migration of CD11b^+^ DCs, supporting our hypothesis (Fig. 7I and J). Altogether, these observations suggest that C3a and C5a produced by CD103^+^ DCs are crucial for their migration to the dLN in order to initiate protective T cells responses and also control the migration of CD11b^+^ DCs.

### Locally produced C3a and C5a provide migratory signals by interacting with their receptors on mDCs

**Figure 8:**
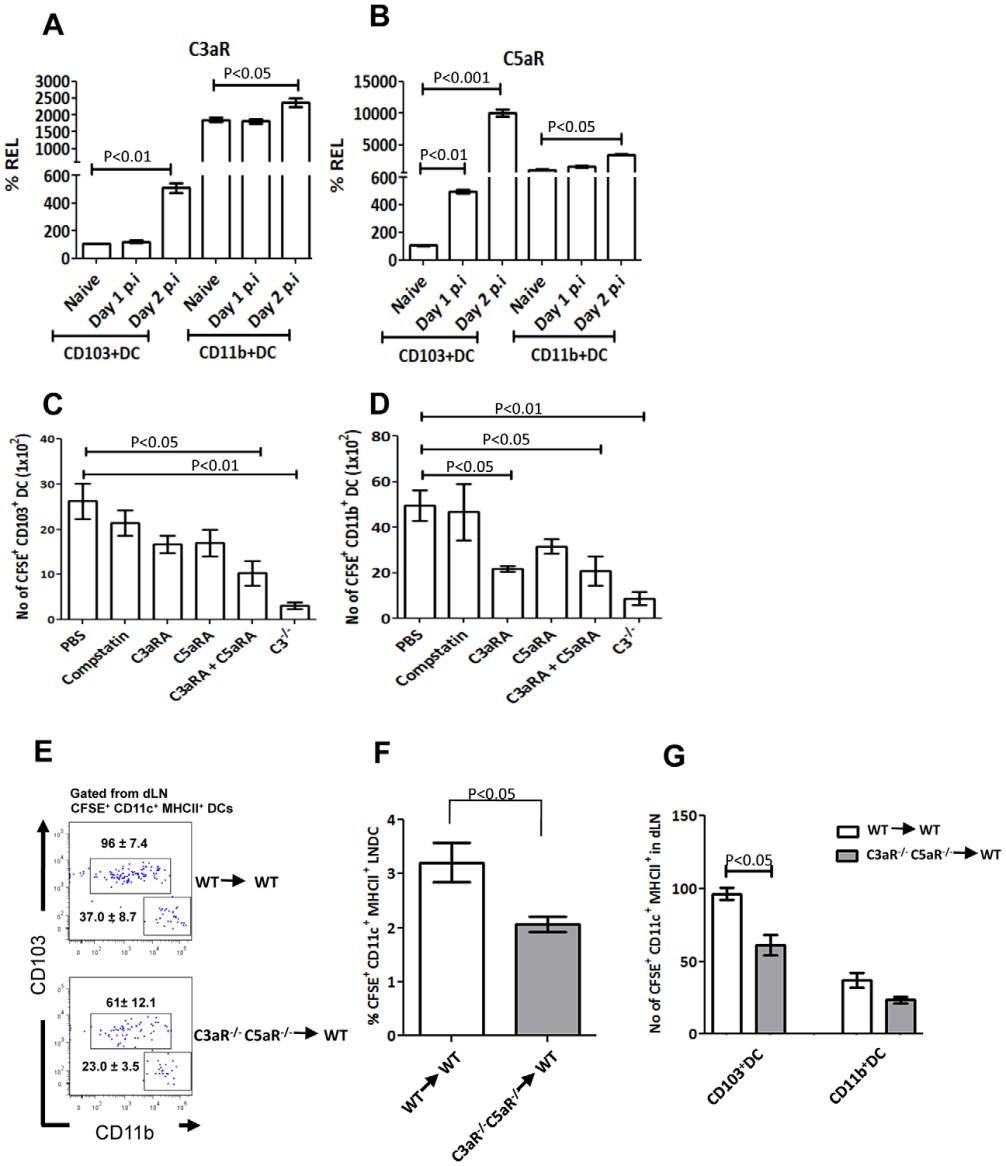
C3aR and C5aR expression on mDCs is upregulated upon influenza infection and blockade of the receptors inhibits mDC migration to the dLN. C3aR (**A**) and C5aR (**B**) expression in FACS sorted lung CD103^+^ DC and CD11b^+^ DC subsets in naïve or PR8 infected WT mice on day 1 and 3 post infection. Highest expression level in a naïve mouse is taken as 100%. (**C** and **D**) Tracking of mDC from the lung to the dLN (as described in Fig. 3) in mice treated with PBS or compstatin or C3aR antagonist (C3aRA) or C5aR antagonist (C5aRA) or both C3aRA and C5aRA together. Bar graphs show the absolute numbers of CFSE^+^ CD103^+^ DC (**C**) and CD11b^+^ DC (**D**) subsets in the draining lymph nodes on day 2 post infection. The data are representative of three different experiments with similar results. The values are expressed as mean ± SEM. (**E–G**) Total CD11c^+^ cells were purified from the lungs WT and C3aR^−/−^C5aR^−/−^ mice that has been flu infected 18 hours before. Purified cells were CFSE labeled and then transferred (5×10^5^ cells obtained by pooling CD11c^+^ cells from 5–6 mice) into a WT mice intratracheally that has been previously (36 hours before) infected with flu. CFSE^+^ mDCs were analysed for the number of CD103^+^ and CD11b^+^ DCs in the dLN of recipient mice 18 hours after adoptive transfer of cells. (**E**) Right panel shows the number of CD103^+^ and CD11b^+^ DCs within the CFSE^+^ mDC population in the dLN. Numbers indicate the number of cells within the gate. (**F**) Percentage of CFSE^+^ mDC in the dLN gated from CD11c^hi^ MHC II^hi^ cells as shown in Fig. S2. (G) Bar graph showing the number of CD103^+^ and CD11b^+^ DCs within the CFSE^+^ mDCs in the dLN of adoptively transferred recipient mice. The data are representative of three different experiments with similar results.

Our data suggest that sensing of complement activation products C3a and C5a by mDCs is crucial for their migration to the dLNs, which could be mediated by expression of the receptors C3aR and C5aR on mDCs during infection. Thus, we examined the expression of C3aR and C5aR mRNA in mDC subsets by qRT-PCR. In CD103^+^ DCs, there was a slight increase in the expression of C3aR mRNA on day1 post infection, and a 4-fold increase by day 2 post infection. C5aR also increased by 4-fold on day 1 and by 90-fold on day 2 post infection (Fig. 8A and B). In CD11b^+^ DCs, the expression of both C3aR and C5aR was higher even under steady state as compared to CD103^+^ DCs and showed a modest increase on day 2 post infection (Fig. 8A and B).

To determine whether mDC migration to the dLNs was directly controlled through interaction of C3a and C5a with their receptors, we blocked C3aR and C5aR either alone or together *in vivo* using specific, high affinity competitive antagonists [32], [33]. Compstatin was used as the control peptide since it is known to possess no complement inhibiting property in mice [33]. Treatment with antagonists started 2 days before infection and was continued daily. CFSE instillation was done 16 hours before sacrificing the mice. At day 2 post infection, the number of CD103^+^ DCs and CD11b^+^ DCs were quantified within the CFSE^+^ mDCs in the dLN. CD103^+^ DC numbers were marginally reduced when either C3aR or C5aR was blocked but were significantly reduced when both were blocked together (Fig. 8C). A significant reduction in CD11b^+^ DC migration was observed when C3aR was blocked alone or together with C5aR (Fig. 8D). These results indicate that direct signaling through both complement receptors C3aR and C5aR is critical for the migration of mDCs from the lung to the dLNs.

To confirm that the C3aR and C5aR receptors on mDCs directly mediate their migration rather than by the receptors on other cells in the lungs, we purified total CD11c^+^ DCs from the lungs of WT and C3aR^−/−^C5aR^−/−^ mice that had been flu infected 18 hours before, CFSE labeled and transferred them intratracheally to flu infected WT recipient mice. We then determined the number of donor CD103^+^ DCs and CD11b^+^ DCs in the dLN 18 hours after adoptive transfer. We observed a significantly reduced CD103^+^ DCs in the mice that received CD11c^+^ DCs from C3aR^−/−^C5aR^−/−^ mice in comparison with mice that received CD11c^+^ DCs from WT mice. Although the CD11b^+^ DCs showed a similar trend, it is important to note that the CD11b^+^ DCs have delayed migration kinetics as compared to CD103^+^ DCs. These results suggest a direct role for these receptors on these mDCs in their migration to the dLN during influenza infection (Fig. 8E–G).

## Discussion

Influenza infection is known to activate complement in the lung with an increase in complement and complement activation products, although the pathway through which it is activated remains unknown [22], [23]. Our study has uncovered the role of lung resident DCs in this process and highlighted their importance as complement producing and sensing cells. Our observations showed that under steady state conditions, lung C3a levels are high and are likely contributed by cells other than CD103^+^ DCs. However, it is interesting to note that upon influenza infection, CD103^+^ DCs exclusively produced high levels of C3 and C5, yielding activation fragments C3a and C5a. These anaphylotoxins, in turn, interacted with their receptors on both CD103^+^ DCs and CD11b^+^ DCs to promote DC migration.

Furthermore, our studies using C3aRA and C5aRA demonstrate that C3a and C5a exhibit overlapping but not fully redundant functions because blockade of both their receptors has a significantly more profound effect on mDC migration than the blockade of either alone. Also adoptive transfer experiments using both receptor (C3aR^−/−^C5aR^−/−^) deficient lung DCs indicated that signaling mediated by both C3a and C5a on mDCs is required for their effective migration. Our results also highlighted the requirement of C3 in the maintenance of CD103^+^ DCs under steady state. Complement is known to regulate DC induced inhalation tolerance through C3's opsonisation property on innocuous antigen [34]. Hence in the absence of C3, innocuous antigens are less efficiently taken up by CD103^+^ DCs, decreasing their ability to migrate (our data shows decreased number mDC in the dLN under steadystate) and thus proliferating less under steady state. However, the requirement of C3 in the maintenance of CD103^+^ DCs was limited to steady state, since under strong inflammatory conditions, deficiency of C3 seems not to affect CD103^+^ DCs number in the lung.

The specific production of C3 and C5 by CD103^+^ DCs underlines their central role in transporting antigen from the lung to the dLN, allowing the priming of T-cells during the early phase of influenza infection [10], [25]. It is also interesting to note that CD103^+^ DCs control the migration of CD11b^+^ DCs based on the fact that CD11b^+^ DCs do not produce C3and C5 upon influenza infection, and that their migratory function is facilitated through the production and generation of complement activation products from CD103^+^ DCs. Thus, to the best of our knowledge, our data demonstrates for the first time how one subset of DC is able to control the functioning of another in a complement dependent manner. Therefore, the defective priming observed in the C3^−/−^ mice may be primarily due to a profound defect in DC-dependent transport of viral antigen to the dLN. In addition the frequency of resident DCs (CD8α^+^ DCs) in dLN were comparable between WT and C3^−/−^ mice (Fig. S2), ruling out their contribution to this defect. This is further supported by our observations in CD103^+^ DC-depleted langerin-DTR mice which showed deleterious effects on survival and effector T cell responses similar to those observed in C3^−/−^ mice upon influenza infection.

Our results also indicated that the percentage of mDCs that express CCR7 was low under steady state in the C3^−/−^ mice and remained low during influenza infection despite CCR7 upregulation. These results suggest that lack of complement affects CCR7 expression only under steady state, but that CCR7 upregulation during maturation was unaffected in the absence of complement mediated signaling (Fig. 5A–C). Although CCR7 has been shown to be critical for mDC migration to the dLN, its upregulation alone was insufficient to compensate for normal mDC migration, suggesting that multiple signals control DC migration to the LNs [35], [36], [37]. Supporting this view, NLRP10, a nucleotide-binding domain leucine-rich-repeat-containing receptors (NLR) has been recently implicated in DC migration, independently of CCR7 mediated signaling [38]. Of note, expression of factor-H and CD59a, genes that control complement activation was found to be altered in DCs from NLRP10^−/−^ mice, suggesting a possible coordination between complement and NLRP10 in facilitating DC migration [38].

Previous studies have shown that DC-derived and -activated C3 and C5 could signal via C3aR and C5aR in an autocrine manner to promote T-cell activation during cognate interaction [19]. In addition, C5aR^−/−^ DCs exhibited attenuated proinflammatory cytokine production, lower expression of MHC-II and costimulatory molecules in response to LPS challenge, as well as reduced capacity for allospecific T-cell stimulation [39]. Similarly, C3-deficient macrophages exhibited lower MHC-II expression and poor ability to expand alloreactive T-cells [40]. These studies, however, were limited by the fact that experiments were performed under *in vitro* conditions and were confined to bone marrow-derived DCs or macrophages. Furthermore, it is not clearly well established how much these *in vitro* derived cells are related to resident DCs within non-lymphoid organs. Tissue mDCs are derived from committed circulating DC precursors which migrate from the bone marrow to the periphery and differentiate into distinct DC subsets [7], whereas bone-marrow DCs were proposed to be rather of monocytic origin [41], [42]. Furthermore, monocyte-derived DCs or inflammatory DCs are absent at steady state and only appear during inflammatory conditions, which permits their differentiation from Ly6C^hi^ blood monocytes [31], [41]. Apart from their late appearance, these monocyte-derived DCs possess little ability to acquire viral particles, although their soluble protein uptake capacity is comparable with mDCs [43]. Furthermore, we observed that C3 does not play a costimulatory role on lung mDCs as the expression of maturation markers (Fig. 1E,F, Fig. 4E and Fig. 5A–C) and the priming ability of mDCs from the dLN were comparable between WT and C3^−/−^ mice during influenza infection (Fig. 1G). Supporting this similarly, the expression of costimulatory molecules on cDCs was not affected after infection with *Listeria monocytogenes* in C3^−/−^ mice [44], suggesting that complement mediated signaling is dispensable for DC maturation during infection. These results are in sharp contrast with previous observations using bone marrow-derived DCs, stimulated with soluble ovalbumin or LPS [19], [20]. It is thus important to draw a distinction between these two DC subtypes as it is apparent that complement signaling may mediate different functions on these cells. We also observed that acute administration of LPS was unable to overcome the defective mDC migration in C3^−/−^ mice suggesting that complement mediated signaling operate independently of the inflammatory signal in mediating lung mDC trafficking during influenza infection.

CD8^+^ T cell responses are critical in the protection against influenza infection [45], and it is noteworthy that C3 deficiency affects T cell immunity, viral clearance and survival [16], [22]. Early lethality in CD103^+^ DC depleted langerin DTR mice paralleling C3^−/−^ mice though surprising, highlights the critical importance of C3 and its contribution by CD103^+^ DCs in the control of early viral replication. C3^−/−^ mice showed increased early viral replication kinetics as compared to WT mice (data not shown) suggesting a role for C3 in mediating innate antiviral immunity. Thus, our observations reiterate the critical importance of C3 during influenza infection, and we surmise increased viral load leading to pneumonia as a possible cause of mortality in the C3^−/−^ mice. This is further strengthened by the report in the recent 2009 H1N1 pandemic patients wherein individuals who had severe disease had lower C3 levels in the serum, while the C3 concentrations were higher in moderately ill subjects [46]. These observations clearly suggest that complement has a key role in determining the outcome of influenza infection in both mice and humans. Because of its crucial role in protection against influenza, defects in many of the complement proteins, although rare, may be associated with increased susceptibility to influenza infection. Strikingly, deficiency or a defect in factor H and factor I are known to increase the susceptibility to bacterial infections, due to lack of C3 regulation [47]. Similarly, deficiency of mannose-binding lectin (MBL), an activator of complement via the lectin pathway, is more prevalent as compared to other complement component deficiency and is known to be associated with increased susceptibility to upper respiratory tract infections in human and to influenza infection in mice [48], [49]. Although complement deficiency is rare in humans, genetic polymorphisms in complement proteins such as factor I, factor B, C3 and factor H are known to affect their availability, activity, and susceptibility to chronic disease conditions [50], [51], [52], [53], [54], [55], [56]. Thus, it is possible that complement deficiency/polymorphisms which affect the level and functioning of C3 in an individual may be associated with higher susceptibility to influenza infection as a consequence of compromised DC migration, T-cell priming and decreased viral clearance resulting in severe disease outcome. By demonstrating a novel role for C3 in regulating tissue DC trafficking, our data may also provide a rationale for complement to be exploited as a target for controlling tissue immunity by modulating mDC emigration and as an adjuvant in novel vaccine strategies for enhancing mDC migration to the lymph node to initiate stronger T-cell responses.

## Materials and Methods

### Ethics statement

Experiments were performed under the approval of the Institutional Animal Care and Use Committee in compliance with the Law and Guidelines for Animal Experiments of the Biological Resource Center (BRC) of Agency for Science, Technology and Research (A*STAR), Singapore. These guidelines were established by the national advisory committee for laboratory animal research as per the Animals and Birds Act 2002.

### Mice

C57BL/6 mice were obtained from the BRC, C3^−/−^ C3aR^−/−^ and C5aR^−/−^ mice were purchased from The Jackson Laboratory. C3aR^−/−^ C5aR^−/−^ double receptor mice were generated by crossing C3aR^−/−^ mice and C5aR^−/−^ mice. Langerin DTR, OT-I Rag1^−/−^ and OT-II Rag2^−/−^ mice (Taconic) were obtained from Mutant Mouse Collection Core Service, Singapore Immunology Network (SIgN), Singapore. Homozygous OT-II Rag2^−/−^ mice were crossed once with homozygous CD45.1 mice (Jax) and the offspring from the F1 generation, referred to hereafter as CD45.1^+^OT-II, were used for adoptive transfer studies. Experiments were generally performed with sex matched mice at 6–10 week of age. Animals were bred under specific pathogen-free conditions at the BRC.

### Influenza virus infection

Influenza virus strain A/PR/8/34 (H1N1) was obtained from National Institute for Medical Research (NIMR, London, UK). Recombinant influenza A/PR/8/34 strains containing the chicken OVA epitope SIINFEKL (PR8 OT-I) and chicken OVA peptide ISQAVHAAHAEINEAGR (323–339; PR8 OT-II) were a gift of P. Thomas (St. Jude Children's Research Hospital, Memphis, TN). Mice were infected intranasally with one of the influenza strains at 15 or 25 plaque forming units (PFU) in 25 µl for female or male mice respectively. The influenza dose was optimized to give ∼20% weight loss in WT mice during the peak of infection and recovery without incidence of mortality. In some experiments, diphtheria toxin was administered intraperitoneally (at 7 ng/g of mouse weight) before and/or during the course of influenza infection for depleting langerin-expressing CD103^+^ DCs. In some experiments LPS was administered intratracheally shortly followed by influenza infection.

### Flow cytometry and cell sorting

Anti-mouse Abs used for FACS analysis were CD3-APC from BD Pharmingen; CD11c- PerCPCy5.5, MHCII-PB, CD103-PE, CD11b-PE-Cy7, B220-APCCy7, CD4-PE, CD86- FITC, CD80-FITC, CD40-APC and CD8-PE-Cy7 from eBioscience; Ly6G/Ly6C-APC, CCR7-APC and IFNγ-APC from Biolegend. For live cell gating, live/dead fixable dye (Molecular Probes, Invitrogen) was used. Lung DCs were identified as low auto fluorescent CD11c^high^, MHC-II ^high^, B220 negative and Ly6G negative cells. Detection was performed using secondary Ab, goat anti-rat-FITC or Chicken-anti-goat-Alexa Flour 647 from Jackson Immuno Research Laboratories, Molecular Probes and Invitrogen respectively. Intracellular staining of IFNγ in CD4^+^ T cells was performed by re-stimulating lung lymphocytes with 1 µM of Influenza virus nucleoprotein MHC-II restricted peptide (311–325, QVYSLIRPNENPAHK) overnight in the presence of brefeldin A (Sigma). Cells were stained for surface markers and fixed with fixation/permeabilization buffer (BD biosciences) before staining for intracellular IFNγ. For enumerating virus specific CD8^+^ T cells, enriched lymphocytes from the lungs were stained with PE labelled H-2D^b^ tetramer with the NP _366–374_ epitope ASNENMETM (Immudex) for 15 min at room temperature followed by staining with the following antibodies: CD3e-APC and CD8 PE-Cy7. For C3aR or C5aR staining, cells were incubated with primary Ab for 30 min, followed by secondary Ab for 30 min. All Ab staining was performed at 4°C. Fc blocking Ab (Anti-Mouse CD16/CD32) was used during all FACS staining. Flow cytometric analysis was performed using BD FACS Canto or BD LSR II and analyzed using FlowJo software (Tree Star, San Carlos, CA). Cell sorting of lung and dLN DCs was performed using MoFlo (Beckman Coulter) and BD FACS Aria.

### Measurement of C3a and C5a in BAL fluid by ELISA

C3a and C5a levels in bronchioalveolar lavage fluid were quantified by a sandwich ELISA as described below. MaxiSorp immuno modules (NUNC) were coated with 100 µl rat anti-mouse C3a or C5a capture antibody (BD pharmingen) at 1 or 2 µg/ml in carbonate buffer pH 9.6 or phosphate buffer pH6.5 (as recommended by the manufacturer) respectively and incubated overnight at 4°C. The plates were then washed three times with wash buffer (PBS with 0.05% v/v Tween 20) and blocked with 200 µl blocking buffer (PBS with 10% FBS), following which the plates were washed 3 times before the addition of 100 µl standards or 1/50–250 diluted BAL fluid and incubated for 2 hours at room temperature (RT). The plates were washed again, and then 100 µl of biotinylated rat-anti mouse C3a or C5a detecting Ab (BD pharmingen) was added at 2 µg/ml concentration to each well (RT, 1 hour). After washing, 100 µl of horseradish peroxidase (HRP) conjugated streptavidin at 1 µg/ml (Jackson Immuno research laboratories) was added and incubated for 30 minutes at RT. Subsequently, the plates were washed and 100 µl TMB substrate solution (Pierce Biotechnology Inc) was added to each well, and incubated in the dark (RT, 30 min). The reaction was stopped with the addition of 50 µl stop solution (2N H_2_SO_4_) to each well. The absorbance was read at 450 nm; reference wavelength 570 nm (Tecan GENios/Magellan). C3a or C5a levels were expressed as ng/100 µg of BAL fluid protein. Total protein in the BAL fluid was quantified by Bradford method (Bio-Rad) following manufacturer's instructions.

### Analysis of T cell proliferation *in vivo* and *ex vivo*

CD4^+^ T cells (from OT-II mice) and CD8^+^ T cells (from OT-I mice) were enriched using MACS beads (Miltenyi Biotec) from spleen and lymph node single cell suspension. Enriched cells were labelled with 5 µM of CFSE violet (Molecular Probes, Invitrogen) following manufacturer's instructions. Approximately 2×10^6^ CD8^+^ T cells or 5×10^6^ CD4^+^ T cells in 200 µl volume were injected retro-orbitally followed by intranasal flu infection. dLN were harvested on day 3 and T cell proliferation was determined by CFSE dilution. For *ex vivo* analysis mDC subsets were sorted from the dLN of OT-I PR8 infected mice and co-cultured in U bottomed plates at a 1∶10 ratio with CFSE labelled OT-I CD8^+^ T cells for 3 days. T cell divisions were measured by flow cytometry. The division index was calculated using Flow Jo software.

### DC migration studies

Influenza infected mice were intranasally instilled with 25 µl 8 mM CFSE (Molecular Probes, Invitrogen) for labelling lung DC *in vivo* at indicated time points. Mice were sacrificed 16 hours later and the dLN were removed and analysed for DCs. In some experiments, mice were treated intraperitoneally with either C3aR antagonist - N^2^-[(2,2-Diphenylethoxy) acetyl]-L-arginine (SB290157, Calbiochem) at 500 µg/mouse or C5aR antagonist- cyclic hexapeptide AcF[OPdChaWR] at 1 µg of peptide/g of mouse weight or both together. Compstatin at a concentration of 1 µg of peptide/g of mouse weight was used as the control. Treatment was started 2 days before influenza infection and continued daily until the mice were sacrificed. In some experiments CD11c^+^ DCs were purified from the pooled lungs of influenza infected mice, CFSE labeled and then administered into the lungs of influenza infected mice. The migration of recipient mDCs in the donor mice were analysed in the dLN 18 hours after adoptive transfer.

### Reverse transcription of mRNA, RT-PCR, and primers

Total RNA was obtained from lung tissue/sorted DC population using the RNeasy kit (Qiagen), following which cDNA was then obtained using QuantiTect Reverse Transcription kit (Qiagen). Both kits were used as per manufacturer's protocol. Real-time PCR was performed on an ABI7500 real time PCR system using SYBR Green (Applied Biosystems) Primers used for qRT-PCR are as follows: Influenza M-protein-forward: 5′-GGA CTG CAG CGT TAG ACG CTT-3′ and reverse: 5′-CAT CCT GTT GTA TAT GAG GCC CAT-3′; C3-forward: 5′-AAG CAT CAA CAC ACC CAA CA-3′ and reverse: 5′- CTT GAG CTC CAT TCG TGA CA-3′; C5-forward: 5′-GCA TTT CTG ACA CCA GGC TTC-3′ and reverse: 5′- AGC GCA CAG TCA GCT TCC A-3′; C3aR-forward: 5′-TGA AAG CAG GGA GTG TTG AG-3′ and reverse: 5′-TGC TCA CTT GCT CAC ATG AA-3′; C5aR-forward: 5′- CCA TGG ACG ACT CCT AAG GT-3′ and reverse: 5′-CTC CTC TAC ACC GCC TGA CT-3′

### Statistical analysis

Data were analyzed using Prism GraphPad software. Statistical significance was determined by one-way ANOVA or the unpaired Student *t* test.

## Supporting Information

Figure S1 **C3^−/−^ mice show decreased effector T cell response and viral clearance upon infection with influenza.** WT and C3^−/−^ mice were infected with flu and on day 7 post infection the flu specific CD4^+^ and CD8^+^ T cell response were evaluated. (A) Graph shows the frequency of IFNγ secreting CD4^+^ T cells in lungs by *ex vivo* overnight stimulation with MHC-II flu peptide on day 7 post infection. (B) Bar graph shows the absolute numbers of IFNγ secreting CD4^+^ T cells in lungs on day 7 post infection (C) Graph shows the frequency of Flu specific CTL response in lung as measured by Flu _peptide_ (ASNENMETM (NP _366–374_)/H-2D^b^ tetramer staining on day 7 post infection. (D) Bar graph shows the absolute numbers of flu specific CD8^+^ T cells in lungs by tetramer staining on day 7 post infection.(E) Graph shows the relative mRNA expression level of influenza M protein in lung tissues of WT and C3^−/−^ mice at the indicated days after influenza infection.
(PDF)

Figure S2 **Characterization of dLN mDC subsets.** Dot plots show the flow cytometric analysis of dLN mDC subsets and resident CD8α DCs in naïve WT and C3^−/−^ mice. Gate III (resident DCs) and IV (mDCs) were selected on the basis of CD11c^hi^ MHC II^hi^ expression. Gate IV were subsequently divided on the basis of CD103 (gate V) and CD11b (gate VI) expression. Gate III were subsequently gated for CD8α DCs (gateVII) on the basis CD8α and CD11b expression
(PDF)

Figure S3 **Characterization of lung mDC subsets.** Dot plots show the flow cytometric analysis of lung DC subsets in naïve WT and C3^−/−^ mice. Autofluorescent cells were excluded from the analysis (gate III) and subsequently plasmacytoid DCs (pDCs) and Gr-1^+^ cells were gated out on the basis of B220 and Ly6C expression (gate IV) respectively. The remaining DCs were defined as CD11c^+^ MHC-II^+^ (gate V) which were further divided on the basis of the expression of CD103 (gate VI) and CD11b (gate VII).
(PDF)

Figure S4 **A. Flow cytometric analysis for the expression of langerin-EGFP on lung DCs and other lineage cells in langerin-DTR mouse.** Single cell preparations from the lungs of WT and langerin-DTR were gated for live cells (DAPI^-ve^) and CD45^+^ve cells and then analyzed for indicated lineage markers. Expression of langerin was analyzed through the expression of GFP (langerin-DTR mice expresses GFP under the control of langerin) on the indicated cell types. Histograms shows the expression of langerin-GFP in the depicted populations. Grey : WT, Open: langerin-DTR mice) **B. Flow cytometry data to show specific depletion of CD103^+^ DCs in the lungs of langerin-DTR mice.** WT and langerin-DTR mice were either treated with DT or not and the number of CD103^+^ and CD11b^+^ DCs in the lungs were evaluated by flow cytometry after 48 hours after DT administration.. Numbers indicate the number of cells within each gate.
(PDF)

Figure S5 **Diphtheria toxin (DT) does not show any toxicity during influenza infection.** (**A**) Percentage of body weight loss after influenza infection. A weight loss of <20% and recovery represents a sub-lethal infection. (**B**) Survival curve comparing influenza infected +/− DT. (n = 6–7 in each group).
(PDF)
