# Supplementary figures and images for: Complement Mediated Signaling on Pulmonary CD103+ Dendritic Cells Is Critical for Their Migratory Function in Response to Influenza Infection
Source: PLoS Pathog. 2013 Jan 10;9(1):e1003115. doi: 10.1371/journal.ppat.1003115 (PMC3542115; doi:10.1371/journal.ppat.1003115)

Figure S1

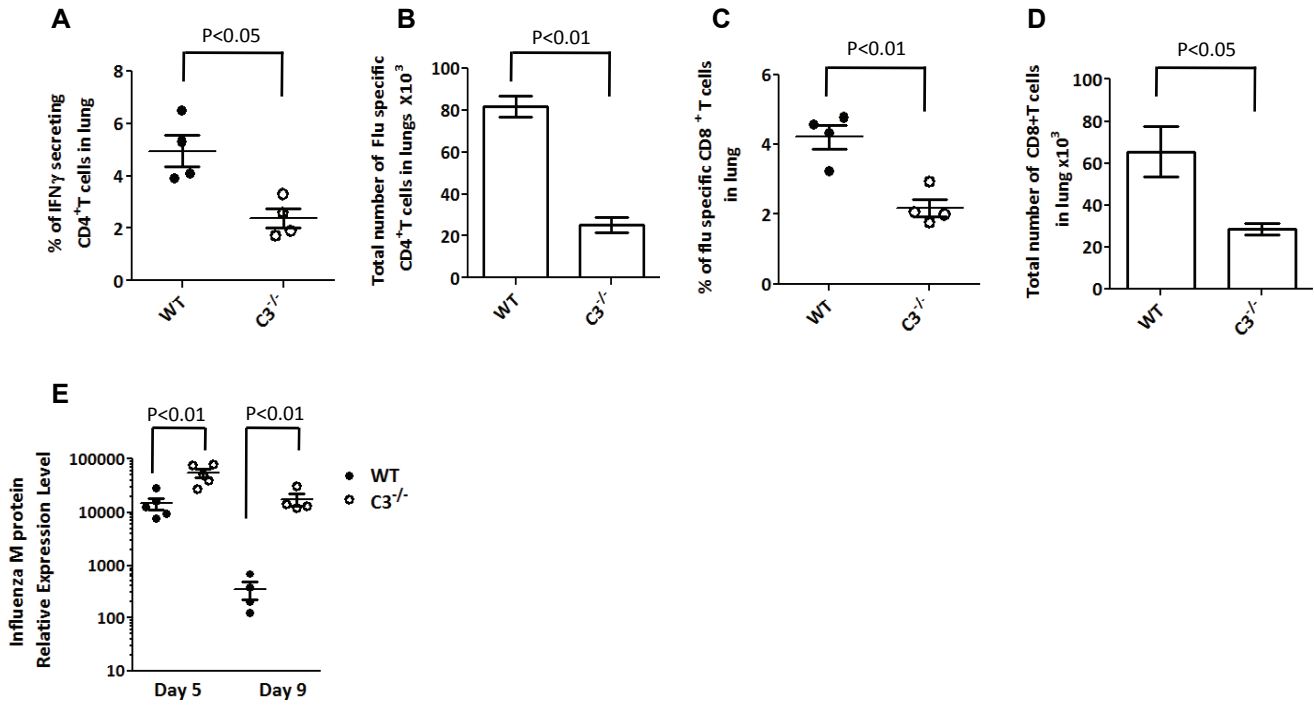

Supplement: Figure S1 — C3−/− mice show decreased effector T cell response and viral clearance upon infection with influenza. WT and C3−/− mice were infected with flu and on day 7 post infection the flu specific CD4+ and CD8+ T cell response were evaluated. (A) Graph shows the frequency of IFNγ secreting CD4+ T cells in lungs by ex vivo overnight stimulation with MHC-II flu peptide on day 7 post infection. (B) Bar graph shows the absolute numbers of IFNγ secreting CD4+ T cells in lungs on day 7 post infection (C) Graph shows the frequency of Flu specific CTL response in lung as measured by Flu peptide (ASNENMETM (NP 366–374)/H-2Db tetramer staining on day 7 post infection. (D) Bar graph shows the absolute numbers of flu specific CD8+ T cells in lungs by tetramer staining on day 7 post infection.(E) Graph shows the relative mRNA expression level of influenza M protein in lung tissues of WT and C3−/− mice at the indicated days after influenza infection. (PDF) [file ppat.1003115.s001.pdf]

**Figure S2**

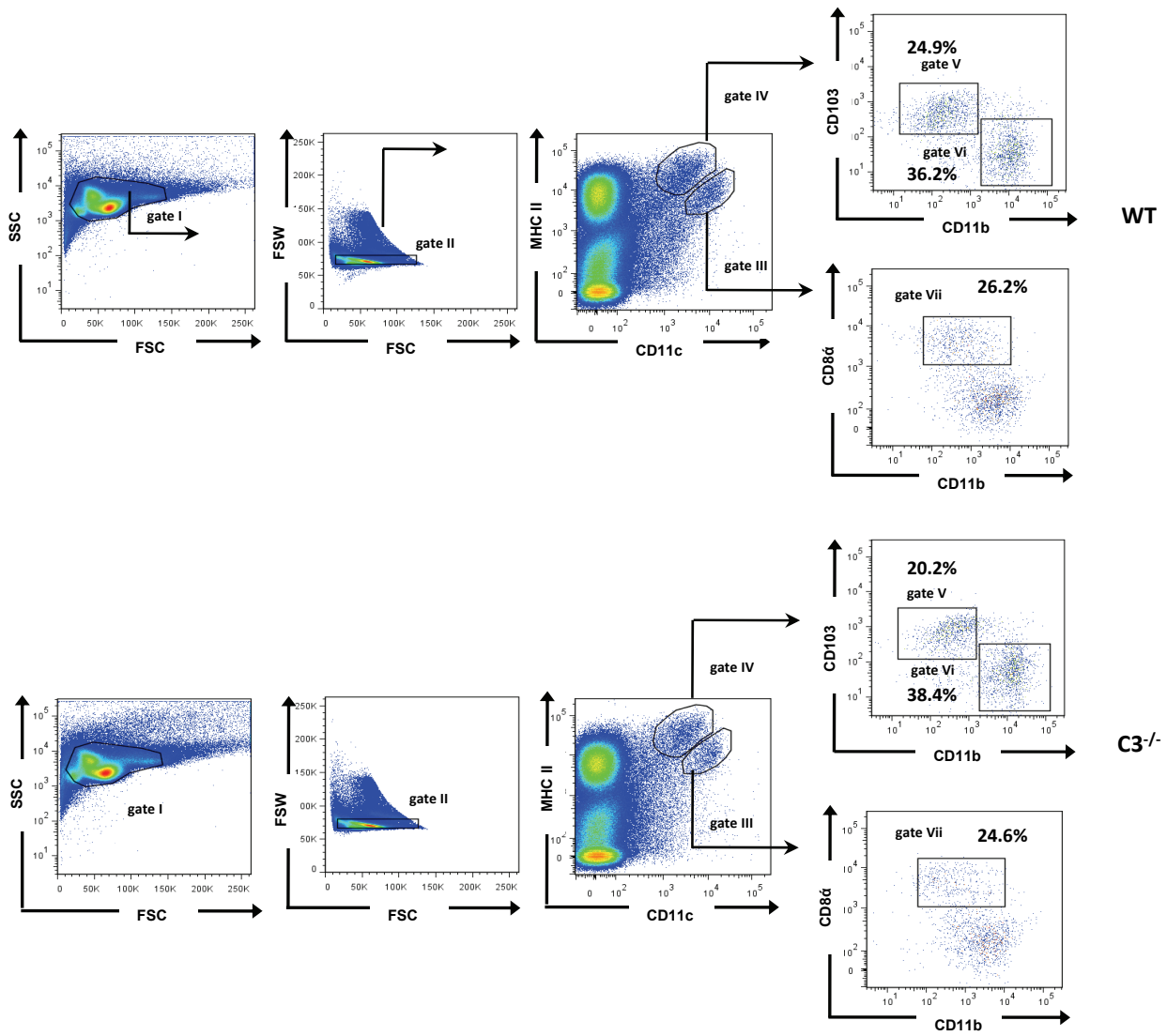

Supplement: Figure S2 — Characterization of dLN mDC subsets. Dot plots show the flow cytometric analysis of dLN mDC subsets and resident CD8α DCs in naïve WT and C3−/− mice. Gate III (resident DCs) and IV (mDCs) were selected on the basis of CD11chi MHC IIhi expression. Gate IV were subsequently divided on the basis of CD103 (gate V) and CD11b (gate VI) expression. Gate III were subsequently gated for CD8α DCs (gateVII) on the basis CD8α and CD11b expression (PDF) [file ppat.1003115.s002.pdf]

**Figure S3**

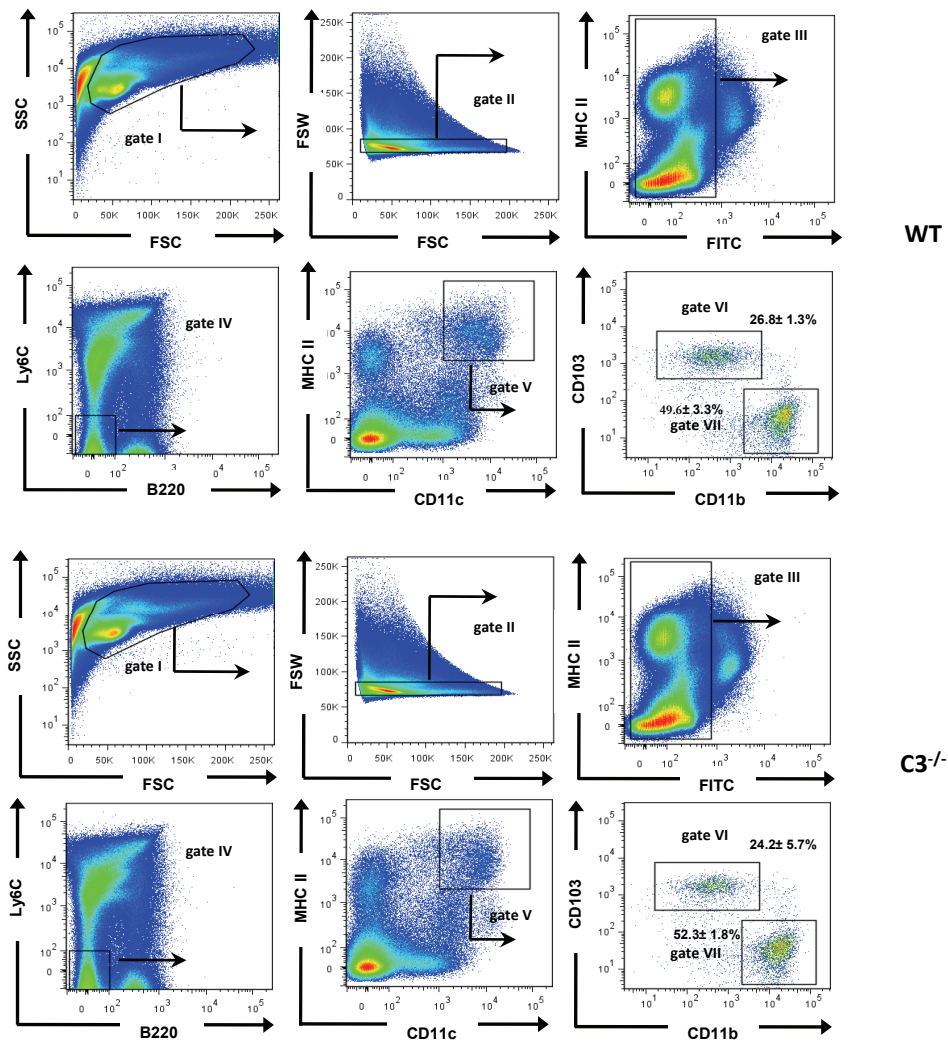

Supplement: Figure S3 — Characterization of lung mDC subsets. Dot plots show the flow cytometric analysis of lung DC subsets in naïve WT and C3−/− mice. Autofluorescent cells were excluded from the analysis (gate III) and subsequently plasmacytoid DCs (pDCs) and Gr-1+ cells were gated out on the basis of B220 and Ly6C expression (gate IV) respectively. The remaining DCs were defined as CD11c+ MHC-II+ (gate V) which were further divided on the basis of the expression of CD103 (gate VI) and CD11b (gate VII). (PDF) [file ppat.1003115.s003.pdf]

**Figure S4**

**A**

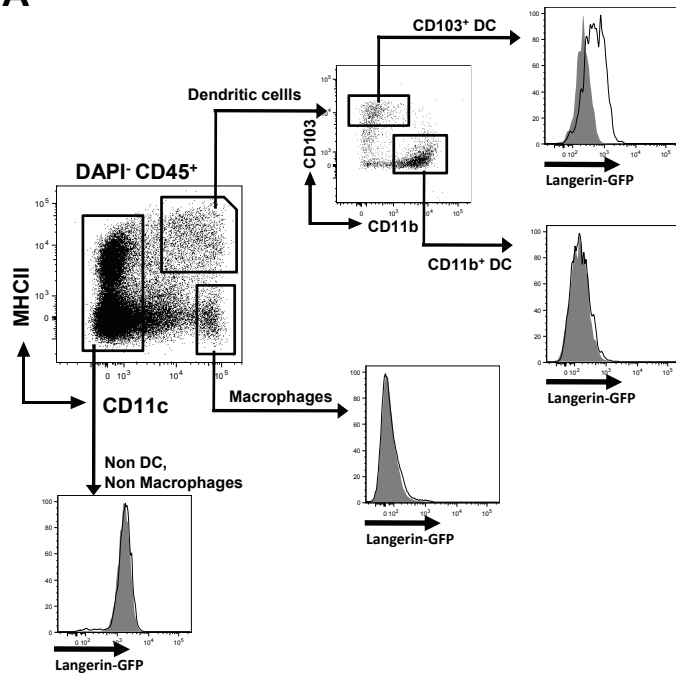

**B**

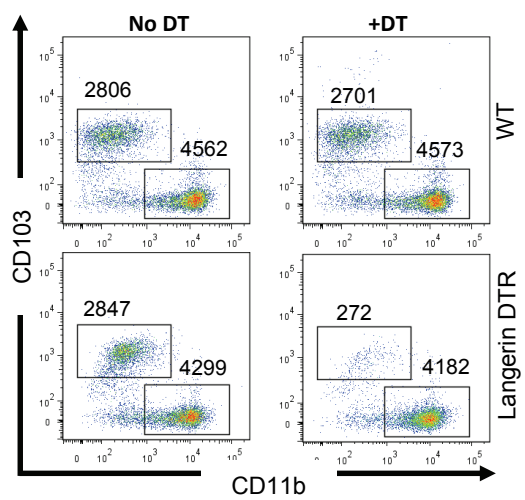

Supplement: Figure S4 — A. Flow cytometric analysis for the expression of langerin-EGFP on lung DCs and other lineage cells in langerin-DTR mouse. Single cell preparations from the lungs of WT and langerin-DTR were gated for live cells (DAPI-ve) and CD45+ve cells and then analyzed for indicated lineage markers. Expression of langerin was analyzed through the expression of GFP (langerin-DTR mice expresses GFP under the control of langerin) on the indicated cell types. Histograms shows the expression of langerin-GFP in the depicted populations. Grey : WT, Open: langerin-DTR mice) B. Flow cytometry data to show specific depletion of CD103+ DCs in the lungs of langerin-DTR mice. WT and langerin-DTR mice were either treated with DT or not and the number of CD103+ and CD11b+ DCs in the lungs were evaluated by flow cytometry after 48 hours after DT administration.. Numbers indicate the number of cells within each gate. (PDF) [file ppat.1003115.s004.pdf]

Figure S5

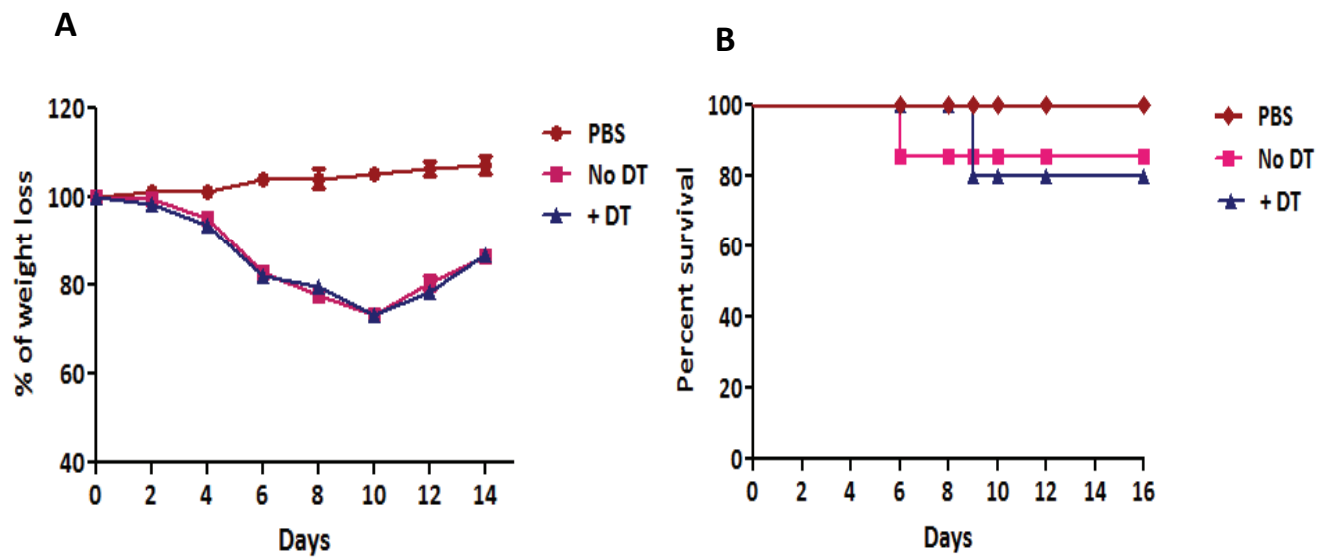

Supplement: Figure S5 — Diphtheria toxin (DT) does not show any toxicity during influenza infection. (A) Percentage of body weight loss after influenza infection. A weight loss of <20% and recovery represents a sub-lethal infection. (B) Survival curve comparing influenza infected +/− DT. (n = 6–7 in each group). (PDF) [file ppat.1003115.s005.pdf]
